# Nanotopography controls cell cycle changes involved with skeletal stem cell self-renewal and multipotency

**DOI:** 10.1016/j.biomaterials.2016.11.032

**Published:** 2017-02

**Authors:** Louisa C.Y. Lee, Nikolaj Gadegaard, María C. de Andrés, Lesley-Anne Turner, Karl V. Burgess, Stephen J. Yarwood, Julia Wells, Manuel Salmeron-Sanchez, Dominic Meek, Richard O.C. Oreffo, Matthew J. Dalby

**Affiliations:** a Centre for Cell Engineering, Institute of Molecular, Cell and Systems Biology, College of Medical Veterinary and Life Sciences, Joseph Black Building, University of Glasgow, Glasgow, G12 8QQ, UK; b Division of Biomedical Engineering, School of Engineering, Rankine Building, University of Glasgow, Glasgow, G12 8LT, UK; c Bone and Joint Research Group, Centre for Human Development, Stem Cells and Regeneration, Institute of Developmental Sciences, University of Southampton, Southampton, SO16 6YD, UK; d Glasgow Polyomics Facility, College of Medical, Veterinary and Life Sciences, University of Glasgow, Wolfson Wohl Cancer Research Centre, Garsube Campus, Bearsden, G61 1QH, UK; e Institute of Biological Chemistry, Biophysics and Bioengineering, William Perkin Building, Heriot-Watt University, Edinburgh, EH14 4AS, UK; f Department of Orthopaedics, Queen Elizabeth University Hospital, Glasgow, G51 4TF, UK

**Keywords:** Nanotopography, Skeletal stem cells, Mesenchymal stem cells, Cell cycle

## Abstract

In culture isolated bone marrow mesenchymal stem cells (more precisely termed skeletal stem cells, SSCs) spontaneously differentiate into fibroblasts, preventing the growth of large numbers of multipotent SSCs for use in regenerative medicine. However, the mechanisms that regulate the expansion of SSCs, while maintaining multipotency and preventing fibroblastic differentiation are poorly understood. Major hurdles to understanding how the maintenance of SSCs is regulated are (a) SSCs isolated from bone marrow are heterogeneous populations with different proliferative characteristics and (b) a lack of tools to investigate SSC number expansion and multipotency. Here, a nanotopographical surface is used as a tool that permits SSC proliferation while maintaining multipotency. It is demonstrated that retention of SSC phenotype in culture requires adjustments to the cell cycle that are linked to changes in the activation of the mitogen activated protein kinases. This demonstrates that biomaterials can offer cross-SSC culture tools and that the biological processes that determine whether SSCs retain multipotency or differentiate into fibroblasts are subtle, in terms of biochemical control, but are profound in terms of determining cell fate.

## 1 Introduction

Adult mesenchymal stem cells are maintained in cellular microenvironments, termed ‘niches’, in the body. These niches provide biochemical and physical cues to the cells to prevent undesired differentiation [1]. We note that more precisely, mesenchymal stem cells from the bone marrow should be described as skeletal stem cells (SSCs), differentiating them from mesenchymal cells from other niche locations [2], [3]. These cells hold osteogenic, chondrogenic, reticular and adipogenic potential and give rise to the haematopoiesis-supportive stroma [4].

Given that SSCs can only be isolated in very small numbers, these cells need to be expanded in culture, away from niche regulation, to achieve numbers sufficient for regenerative therapies/tissue engineering. Therefore, understanding the control of SSC self-renewal is equally important as understanding the mechanism of differentiation. However, to date, the mechanisms that control SSC self-renewal remain elusive. For example, there has been only one report that illustrates that SSC adhesion/cytoskeletal morphologies are subtly different to that of the fibroblast, both with intermediate intracellular tension compared to adipocytes and osteoblasts [5], [6]. The report highlighted that SSCs displayed a slightly lower tension phenotype than fibroblasts [7]. Indeed SSCs and fibroblasts look morphologically similar, with SSCs originally described as fibroblastic progenitors/fibroblast colony forming units [8], [9]. It is therefore likely that these close similarities between SSC and fibroblast morphologies means that biochemical regulation controlling the SSC and fibroblast phenotypes is subtle even though the phenotypical differences are large. Failure to control these small differences in culture likely results in the fibroblastic over-growth typically seen *in vitro*. Therefore an, as yet, unmet need remains to develop methods that will enhance SSC self-renewal (population expansion without loss of multipotency) [10], [11], while preventing spontaneous differentiation of SSCs to fibroblasts when propagated in cell culture.

Changes in chemistry [5], [6], [12], [13], stiffness [14], [15], [16] and nanoscale topography [17] of cell culture growth surfaces have all been used to demonstrate that alterations in the physical material characteristics of the cell growth surface can exert control over SSC self-renewal in culture; these materials may therefore provide useful tools to investigate SSC function. Of these, it has been demonstrated that nanopatterned surfaces can preserve long-term multipotency of cultured SSCs [10], [18]. The surface retains SSC marker expression and functional multipotency in prolonged culture and allows retention of marker expression with serial passage in culture [10], [18]. The nanopattern consists of 120 nm diameter features that are of similar size to fenestrae of sinusoidal capillaries, where SSCs are thought to reside in the perivascular stem cell niche [18].

We have used this nanotopographic surface in this report in order to understand the control of SSC proliferation and note that when employing such cell-instructive materials, it appears that cell density can affect SSC morphology and differentiation; *e.g.* lower seeding densities promote osteogenesis and higher seeding densities are permissive for adipogenesis [6].

In this report, in the absence of one accepted defined homogenous SSC population, we start with a focus on ensuring our nanotopographical platform is robust across several subtypes of bone marrow-derived SSCs. That advanced technologies work as platforms across stem cell populations, so that a technology can be termed ‘pan-SSC’, for example, is clearly important so that technologies can translate between labs and find real-world use; yet this important facet is poorly studied.

We have previously demonstrated the prolonged self-renewal (marker retention, functional multipotency) of SSC enriched populations selected for using the trypsin-resistant cell surface marker STRO-1 [19], [20], [21], [22] as well as with commercially available SSCs selected for multiple markers [10]. Here, we expand on these studies to include the widely used enrichment marker, CD271 [23] (also known as low affinity nerve growth factor receptor (LNGFR) or p75NTR, a member of the low affinity neurotrophin receptor and tumor necrosis factor receptor superfamily), which is considered to select SSCs. To optimise SSC self-renewal we first examined importance of seeding densities to derive a standardised protocol in order to allow us to determine the importance of cell cycle regulation for SSC maintenance, hypothesising that mitogen activated protein kinases (MAPKs) would act as a switch from self-renewal to growth.

## 2 Materials and methods

### 2.1 Production of materials

Two master substrates were fabricated on silicon coated with 100 nm polymethylmethacrylate (Elvacite 2041, Lucite International) by electron beam lithography to generate arrays of 120 nm diameter pits with 300 nm centre-centre spacing and 100 nm depth in a square (SQ) arrangement, or arrays with an upto ± 50 nm offset of pits for from the centre position creating a near square (NSQ) arrangement. The silicon substrates were exposed to a 50 kV electron beam (Vistec VB6 UHR EWF electron beam lithography tool), developed in 1:3 methyl isobutyl ketone: isopropyl alcohol for 30 s, rinsed in isopropyl alcohol and finally dried in a nitrogen stream. Nickel dies were made from the patterned resists and 50 nm of Ni-V was sputtered coated. Electroplating was carried out to a thickness of approx. 300 μm (outsourced to DVDNorden, Denmark). These nickel shims were cleaned with chloroform for 10–15 min in an ultrasonic bath, subjected to further rinses in acetone and isopropyl alcohol, and dried once more in gaseous nitrogen. Polycarbonate (Makrolon^®^ OD2015) substrates were generated by injection moulding using an Engle Victory 28 hydraulic injection moulder. The required nickel inlay corresponding to a SQ arrangement or NSQ arrangement was inserted prior to production. Heating to 180 °C melted the polycarbonate. A clamping force of 250 kN was used to imprint onto the surface of the polycarbonate, with the final dimensions of each substrate being 24 mm × 24 mm. The temperature was allowed to drop to 70 °C before separation of the press and polymer. Unpatterned (flat) substrates were used as controls.

### 2.2 Atomic force microscopy

AFM experiments were performed with a Nanowizard 3 (JPK, Berlin, Germany). The images were acquired in tapping mode in air using silicon cantilevers (FMV from Bruker AFM Probes, Billerica, MA) with a pyramidal tip, a force constant of 3 N/m, a resonance frequency of ∼75 kHz, and a radius of curvature below 10 nm. Height, phase and amplitude magnitudes were recorded simultaneously for each image.

### 2.3 Extraction and selection of SSCs

SSC populations were selected by magnetic separation (STRO-1 or CD271) from adherent mononuclear cell fractions from human bone marrow obtained during routine knee/hip replacement surgeries. Briefly, bone marrow aspirate was thinned with basal media (DMEM supplemented with 10% (v/v) FBS, 1% (v/v) sodium pyruvate, 1% (v/v) non-essential amino acids, 1% (v/v) penicillin-streptomycin, 1% (v/v) l-glutamine, 5% (v/v) Fungizone^®^ amphotericin B), and layered onto Ficoll Paque Premium density gradient media (GE Healthcare). Following centrifugation at 1513*g* for 45 min, the intermediate interface of mononuclear cells was removed and washed with media successively for a total of three washes. The resulting pellet was transferred into a tissue culture flask and cells were cultured to near confluence. These were then detached and selected for the marker CD271 using a human CD271 positive selection kit and magnet (EasySep, Stem Cell Technologies) as per the manufacturer's instructions. STRO-1^+^ SSCs were selected similarly using an in-house STRO-1 antibody (hybridoma courtesy of Dr Beresford, University of Bath) using a MACS kit from Miltenyi Biotech. Over the course of this study, cells from 12 donors were used with each biological test using new cells.

### 2.4 SSC culture and seeding

All cells were maintained in DMEM basal media (as outlined in the previous section) at 37 °C and 5% CO_2_. Cell detachment was performed with 1x TrypLE™ select solution (Gibco, Life Technologies) for SSC selection and seeding. Even distribution of cells at the required densities was achieved through the use of a polycarbonate seeding device (patented by Riehle, Gadegaard and Reynolds, University of Glasgow, application number PCT/GB2013/052776). Each sterilised seeding device was placed on top of a substrate, and a cell suspension at the required concentration was pipetted through the entry port incorporated into the seeder design. After several hours, attachment and initial spreading of cells was verified visually by microscopy, prior to removal of the device and further addition of media.

### 2.5 SSC synchronisation

SSCs were seeded onto substrates using seeding devices and allowed to attach for 24 h. Synchronisation in G0 stage was achieved by culture in low serum media (0.1% (v/v) FBS with supplemented sodium pyruvate, non-essential amino acids, penicillin-streptomycin, Fungizone-amphotericin B and l-glutamine in quantities described for maintenance media) for 48 h. Release of SSCs back into cycle was carried out by replacement of low serum media with maintenance media containing 10% (v/v) FBS.

### 2.6 Flow cytometry

Cells corresponding to each patient were washed with PBS, detached and pooled according to substrate type, and culture condition (synchronised only/synchronised with release into the cycle) and then analysed by fluorescence-activated cell sorting (FACS). Cells were pelleted at 345*g*, and subjected to further washing with PBS with 0.1% BSA in ice-cold molecular grade ethanol (70%) was then added whilst simultaneously agitating the cells in the vessel. The resuspended cells were held at 4 °C for 1 h for fixation. Propidium iodide staining solution (3.8 mM sodium citrate, 40 μg/ml propidium iodide in PBS) was added in parallel with 10 μg/ml RNase A for 3 h at 4 °C. Cell cycle distribution was analysed using a Guava easyCyte™ flow cytometer (Millipore), with post-analysis using FLOWJO version 10 software (TreeStar, San Carlos, CA).

### 2.7 Western blotting

Cells cultured on nanotopographies were detached with trypsin-versene, washed and pelleted. Protein was extracted with RIPA buffer (150 mM NaCl, 50 mM Tris-HCl pH 7.5, 1% Triton X-100, 1% deoxycholic acid, 1% SDS) with protease and phosphatase inhibitors added (complete mini, EDTA-free protease inhibitor cocktail and PhosSTOP phosphatase inhibitor cocktail, Roche), equalized after performing a Bradford assay (Biorad), and loaded onto a 4–12% polyacrylamide precast gel after addition of loading buffer (NuPAGE) and heating to 95 °C for 5 min. Gels were run at 200 V for approximately 45 min. Protein transfer was achieved using a PVDF membrane prepared according to the manufacturer's instructions (Amersham Bioscience). MOPS SDS running buffer and transfer buffer were prepared from stock solutions (NuPAGE). The membrane was blocked in 1% (v/v) non-fat milk in PBS for 1 h. Primary antibodies (rabbit monoclonal anti-osteopontin antibody (EPR3688) (Abcam), mouse monoclonal anti-alpha-tubulin antibody IgG1 (clone B-5-1-2) (Sigma), STRO-1 (sc-4773) (Insight Biotech) and CD271 (Insight Biotech)) were incubated with the membrane overnight at suitable dilutions in 1% (v/v) non-fat milk at 4 °C. HRP-linked secondary antibodies (Sigma) were incubated with the membrane for 1 h (1:5000 dilution in 1% (v/v) non-fat milk). Detection was achieved using standard chemiluminescence methods, and visualized using x-ray film.

### 2.8 Immunostaining

Substrates were washed with PBS and cells were fixed in a solution of 4% paraformaldehyde for 15 min at 37 °C. Fixed cells were briefly permeabilised with permeabilisation buffer (50 mM NaCl, 30 mM sucrose, 3 mM MgCl_2_·6H_2_O, 20 mM HEPES, 0.5% v/v Triton X-100 in PBS, pH 7.2) and blocked in 1% (v/v) BSA in PBS for 15 min at 37 °C prior to application of primary antibodies (STRO-1 (sc-4773) (Insight Biotech) and CD271 (Insight Biotech)). All antibody dilutions were made in 1% (v/v) BSA. Primary antibody incubations took place for 1 h at 37 °C at the required dilutions together with a 1:500 dilution of rhodamine-phalloidin (Molecular Probes), after which three washes with 0.5% (v/v) PBS-Tween (PBST) were used to remove any excess. Biotinylated secondary antibodies (Horse biotinylated anti-mouse IgG (1:50; Vector Laboratories)) were diluted 1:50 and incubated with samples for 1 h at 37 °C. Further washes were carried out prior to application of FITC-conjugated streptavidin probes (Vector Laboratories) diluted 1:50 (30 min at 4 °C). After a final three washes, samples were mounted with immunomount solution containing DAPI to stain cell nuclei (Vectorshield, Vector Laboratories).

### 2.9 CellProfiler image analysis

Fluorescence images were analysed using CellProfiler (Broad Institute, USA) open source cell image analysis software (version 2.1.0), which allowed for the quantification of proteins of interest in cells after 28 day culture. Multi-step pipelines incorporating input, processing, threshold and quantification steps allowed the location of cells to be defined from cell nuclei (DAPI staining), and edges of cells to be defined with F-actin staining. Integrated intensity measurements were then obtained for multiple cells within each image.

### 2.10 In-cell western (ICW)

Samples fixed in 4% paraformaldehyde were permeabilised as described for immunostaining and blocked for 1.5 h in Odyssey^®^ blocking buffer (LI-COR). Primary antibodies (for a protein of interest and a normalizing protein) were diluted in tandem in blocking buffer and incubated with the samples overnight at 4 °C. These were washed with 0.1% (v/v) PBST three times, and then incubated with fluorescent-tagged IRdye secondary antibodies (1:800 dilution) for 1 h. After three further washes in PBST, the samples were imaged using Odyssey^®^ Sa Infrared Imaging system (LI-COR). Primary antibodies were all from Cell Signalling as follows: pERK (phospho-p44/42 MAPK (Erk1/2) (Thr 202/Tyr 204) (E10) mouse monoclonal antibody (IgG1), ERK1/2 (p44/42 MAPK (Erk1/2) antibody) rabbit polyclonal, cdc2 rabbit polyclonal antibody, p27 kip 1 rabbit polyclonal antibody, E2F-1 rabbit polyclonal antibody P-Rb (Phospho-Rb (S807/811)), rabbit polyclonal antibody, Cyclin D1 (Cyclin D1 (DCS6)) mouse monoclonal antibody (IgG2a). If a normalizing protein was not used, CellTag (LI-COR) was used to normalize for cell number.

### 2.11 Next-generation sequencing (RNAseq)

Cells were removed from substrates using trypsin-versene solution followed by cell scraping. Cells in suspension were pelleted at 345*g* and washed with PBS to remove residual media. RNA extractions were performed with the RNeasy extraction kit (Qiagen) following the manufacturer's instructions. RNA concentrations were obtained using a Nanodrop ND1000 spectrophotometer (Thermo Scientific) and samples were submitted to the Glasgow Polyomics facility (University of Glasgow). A TruSeq^®^ stranded mRNA low throughput kit (Illumina) was used to process 136 ng of RNA/sample. A library of template molecules for sequencing was generated by conversion of mRNA. The steps included poly-A selection, first and second strand cDNA synthesis, cleanup, adenylation of 3′ ends, ligation and cleaning of adapters, PCR amplification (98 °C for 30s, 15 cycles of: 98 °C for 10 s, 60 °C for 30 s, 72 °C for 30 s, 72 °C for 5 min) with a final cleanup stage. Cleanup was achieved using AMPure XP beads (Beckman Coulter). Sequencing was carried out by adding 3.2 pM libraries (Illumina NextSeq 500 sequencer). Initial bioinformatics was performed using a BaseSpace^®^ next-generation sequencing platform (Illumina), with differential expression data being analysed with Ingenuity Pathway Analysis (IPA) software (Qiagen).

### 2.12 Metabolomics

CD271^+^ SSCs were cultured for 7 days only and STRO-1^+^ SSCs were cultured for both 7 and 28 days to expand the analysis further. Cells were washed with cold PBS prior to carrying out the extraction procedure. Metabolites were extracted using cold extraction solvent (1:3:1 ratio of chloroform:methanol:water). Substrates were placed, cell side down onto a small volume of solvent, within culture plates and sealed. These were agitated continuously for 1 h on a platform shaker at 4 °C. The resulting extracts were centrifuged for 3 min at 13,000*g* to pellet cell debris, and the supernatant kept at −80 °C until ready to proceed with liquid chromatography mass spectrometry (UltiMate 3000 RSLC with a 20 × 2.1 mm ZIC-pHILIC column running at 300 μl/min coupled to an Orbitrap Q-Exactive (Thermo Fisher). A pooled sample was made to assist with quality control. The raw mass spectrometry data was processed as previously described [24], using XCMS for peak picking, MzMatch for pre-processing (grouping and filtering) and IDEOM for post-processing (further filtering and identification). A panel of known metabolites were used as standards, and validated by mass and retention time. Putative identifications of other metabolites were made based on data pertaining to mass and predicted retention time. Differences in metabolome expression and associations to signaling pathways were further investigated using IPA software (Qiagen).

### 2.13 Statistics

Unless otherwise stated, statistics were analysed using ANOVA with Kruskal-Wallis test and Dunn's post-test as we assumed data did not follow normal distribution.

All raw data can be found at: 10.5525/gla.researchdata.341.

## 3 Results

### 3.1 Determining the optimal seeding density for nanotopography-induced SSC growth

**Fig. 1:**
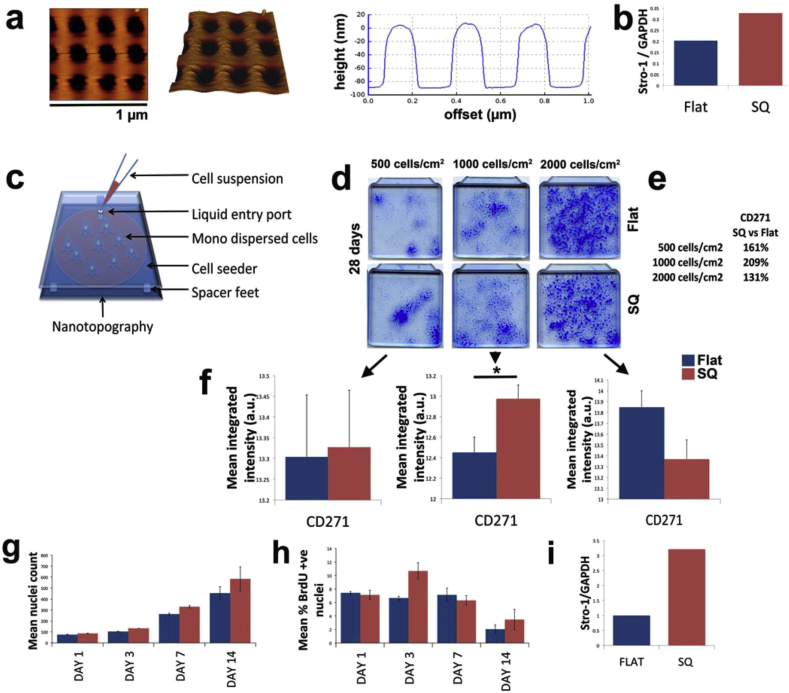
Determining the optimal seeding density for SQ nanotopography. (a) AFM image representative of our self-renewal promoting nanotopographical pattern; the surface of a SQ substrate produced by injection moulding. Dark regions indicate the location of the pits. The graph to the right of the images shows the depth and spacing of the features. Scale bar: 1 μm. (b) STRO-1 protein levels in SSCs cultured on SQ nanotopographies using a conventional seeding method (suspension method at ∼ 3000 cells/cm^2^). Retention of STRO-1 was demonstrated with western blot densitometry after 7 days of culture. Results represent relative density of STRO-1 protein normalised to GAPDH, expressed in arbitrary units (a.u.). (c) Schematic of a seeding device for even cell distribution. Each sterile seeder (produced in-house) was placed on top of the polycarbonate substrate. (d) Full substrate images of SSCs stained with Coomassie Blue after 28 days culture. Distinct growth patterns were observed that appeared associated with initial seeding density. At the low and mid-density conditions (500 cells/cm^2^ and 1000 cells/cm^2^ respectively), growth of cells were mostly confined to colonies (clonal growth), whereas at the highest seeding density (2000 cells/cm^2^), cells spread throughout the surface (fibroblastic growth) reaching full confluence. These observations were not restricted to the SQ nanotopography, and was thus a substrate-independent effect. Representative images shown (n = 3). (e) Percentage of CD271 protein expression at each tested seeding density as a function of the flat control. The data summarises western blot densitometry results (CD721 normalised to GAPDH) following 28 days culture of CD271^+^ SSCs. It can be seen that the highest CD271 stem cell marker levels are found when utilising an initial seeding density of 1000 cells/cm^2^. Each of the other densities tested resulted in a lower percentage of CD271. (f) Levels of CD271 protein in individual cells after 28 day culture were assessed across different seeding densities to provide detailed analysis using CellProfiler image analysis. This involved quantifying integrated intensity levels for each cell across multiple fluorescence images (>40). The data reflects the trends observed with western blotting, in that 1000 cells/cm^2^ gave the highest CD271 readings on SQ relative to flat. At 500 cells/cm^2^, CD271 was similar across the two substrate types whereas at 2000 cells/cm^2^, CD271 was not effectively retained on SQ. *p < *0.05*, ANOVA (mean ± SEM, a.u. = arbitrary units). (g) Assessment of growth (mean nuclei count ± SEM) showed that CD271^+^ SSCs increased in number on SQ as time progressed (n = 3). (h) BrdU staining (mean % BrdU positive nuclei ± SEM) indicated a comparable degree of proliferation between flat and SQ (n = 3). (i) Western blot densitometry (STRO-1 normalised to GAPDH) showing that by seeding STRO-1^+^ SSCs at 1000 cells/cm^2^ on SQ, the effect of stem cell marker retention was reproducible. (For interpretation of the references to colour in this figure legend, the reader is referred to the web version of this article.)

In this report, we use a nanotopographical pattern that supports SSC self-renewal without promoting differentiation; previously it was shown to retain SSC phenotype for STRO-1 selected and purchased SSCs (Promocell GmbH) at ∼ 3000 cells/cm^2^ [10]. This is a pit array, originally patterned using electron beam lithography, consisting of 120 nm diameter nanopits in a square arrangement (300 nm centre-centre spacing) with 100 nm depth - this is denoted as SQ (square). After conversion to a metal shim and injection moulding against this shim into polycarbonate, the replicas have a 120 nm diameter and 80 nm depth feature size with the 300 nm centre-centre spacing retained (Fig. 1a).

To briefly confirm retention of SSC phenotype [10], we used densitometry of western blots probed with an anti-STRO-1 antibody for STRO-1 selected SSCs seeded at 3000 cells/cm^2^, showing more STRO-1 is expressed in SSCs on the SQ surface compared to flat control over a 7 day culture period (Fig. 1b). This supports previous observations [10] and allows us to move on to trial the topography with the CD271^+^ SSC sub-population to better understand the flexibility of the platform.

We therefore repeated this experiment for a longer, 28 day culture with CD271^+^ SSCs to provide a more robust phenotypical analysis. For these experiments, we used the initial seeding density of 3000 cells/cm^2^ with a typical seeding protocol involving adding a suspension of SSCs directly into a culture well containing the substrate. It was found that the topographical effect on maintenance of CD271 expression was negligible with CD271 expression being lost suggesting fibroblastic overgrowth (data not shown). This supports the concept that not all proposed SSC populations are the same and suggests that a high degree of stringency is required to enable SSC technologies to work as platforms for all SSC subpopulations [2]. This led us to devise a robust cell seeding protocol, based on observation that maximising cell-material interaction is important [5], [6], [15], that can be used to reproducibly maintain different SSC sub-populations in cell culture.

As discussed, previous reports have indicated that reducing cell-cell contact and maximizing cell/material interaction is critical to gain optimal control of SSC phenotype using biomaterials strategies [5], [6], [15]. We therefore employed an in-house fabricated cell seeding device (Fig. 1c) that ensures even cell distribution and tested a range of seeding densities; 500, 1000 and 2000 cells/cm^2^. Varying cell growth patterns were observed at these different cell densities (Fig. 1d). At 500 cells/cm^2^ only clonal expansion (intense blue areas) was observed and, after 28 days, the culture was far from confluence suggesting that this seeding density was too low (Fig. 1d). At 1000 cells/cm^2^, both clonal and individual cell expansion was observed with more of the surface covered in cells (Fig. 1d). At 2000 cells/cm^2^, however, large amounts of individual cell growth could be seen and the surfaces were fully confluent (Fig. 1d).

Western blot densitometry (Fig. 1e – cell population observation from lysate) and CellProfiler [25] (Fig. 1f – allowing per cell observation) image analysis for CD271 expression after 28 days of culture indicated that (i) there was negligible difference in expression at 500 cells/cm^2^, illustrating that with purely clonal expansion, substrate type was less important and (ii) at 1000 cells/cm^2^ significantly more CD271 was expressed in SSCs on the SQ surface suggesting that the nanotopography had a strong influence on SSC self-renewal at this cell density. (iii) At 2000 cells/cm^2^, no statistical difference was noted, indicating that cell-cell interaction was overriding the nanotopographical cues. It was seen that the CD271^+^ cells showed normal cell number expansion (*i.e.* similar to cells on planar control, Fig. 1g) and proliferation characteristics (Fig. 1h) at the optimal, 1000 cells/cm^2^, density. Further, western blot densitometry of STRO-1 selected SSCs at this concentration demonstrated retained STRO-1 after 28 day culture (Fig. 1i).

**Fig. 2:**
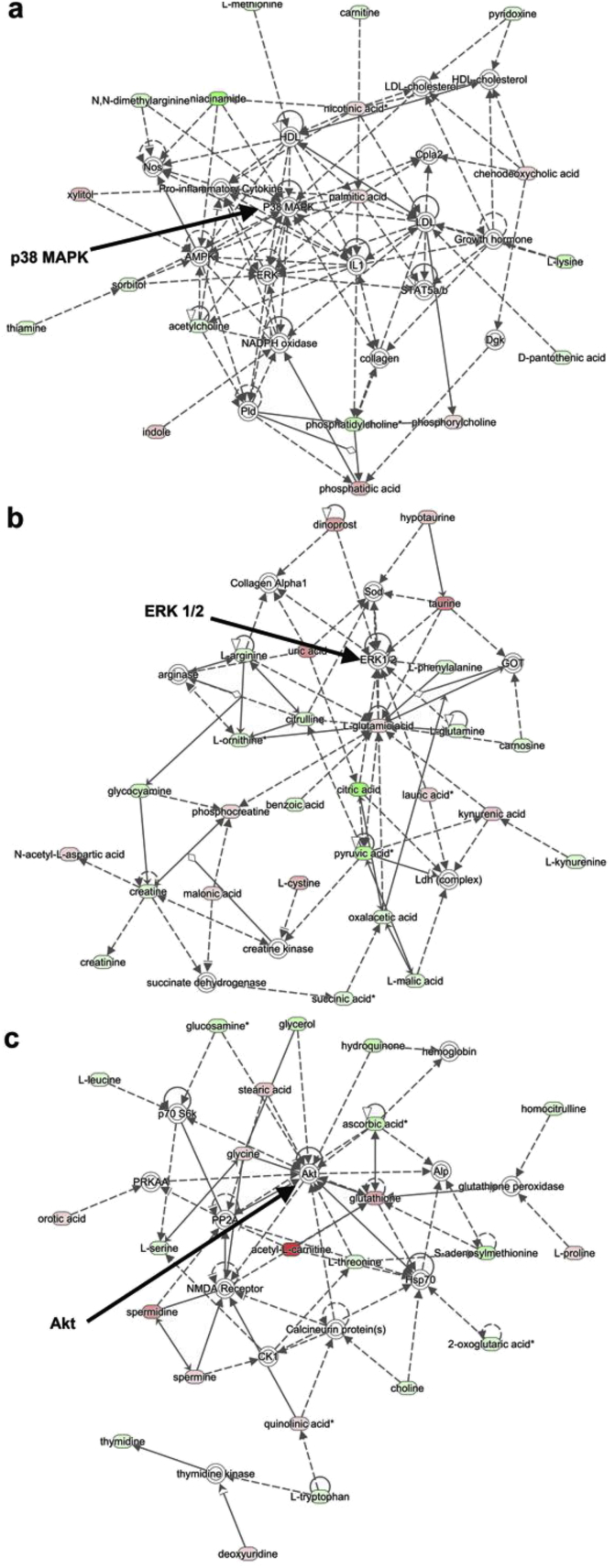
Linking changes in the metabolome to biochemical networks. MS-metabolomics analysis of CD271^+^ SSCs (7 day culture) highlighted that the top networks on SQ with reference to flat controls had associations with (a) p38 MAPK (b) ERK 1/2 and (c) Akt. Contributions to these signalling hubs came largely from metabolites involved in amino acid biosynthesis, glycolysis and nucleotide biosynthesis. Network maps were generated using IPA (n = 6).

To test the impact of our new seeding protocol on CD271^+^ SSCs in culture, metabolomics analysis was carried out. Here, we use analysis of metabolites by mass spectrometry after 7 days of culture for CD271^+^ SSCs cultured on SQ *vs* flat control with a seeding density of 1000 cells/cm^2^ and Ingenuity Pathway Analysis (IPA) to indicate pathways where metabolomic changes link into cellular biochemistry. This analysis was chosen to see if we could infer MAPK regulation without ignoring other possibilities through selection bias. Indeed, the top three networks selected were all related to cell proliferation and tied into p38 MAPK, extracellular signal related kinase, ERK 1/2 (MAPKs) and Akt (a serine/threonine-specific protein kinase) signalling hubs with contributions largely from amino acid biosynthesis, glycolysis and nucleotide biosynthesis, *i.e.* protein and energy metabolite pathways as would be expected to be implicated in control of SSC phenotype [7], [26] **(**Fig. 2**)**.

This provides further confidence that the SQ topography is exerting its self-renewal effect during proliferation in the more cell number sensitive CD271^+^ SSC population and that our optimised cell seeding protocol has a significant effect on retaining SSC phenotype during expansion in extended cell culture. Thus, we can be satisfied that our platform is valid for multiple SSC sub-populations after adjusting seeding conditions.

### 3.2 Differences in cell cycle regulation between SSCs undergoing self-renewal and osteogenesis

**Fig. 3:**
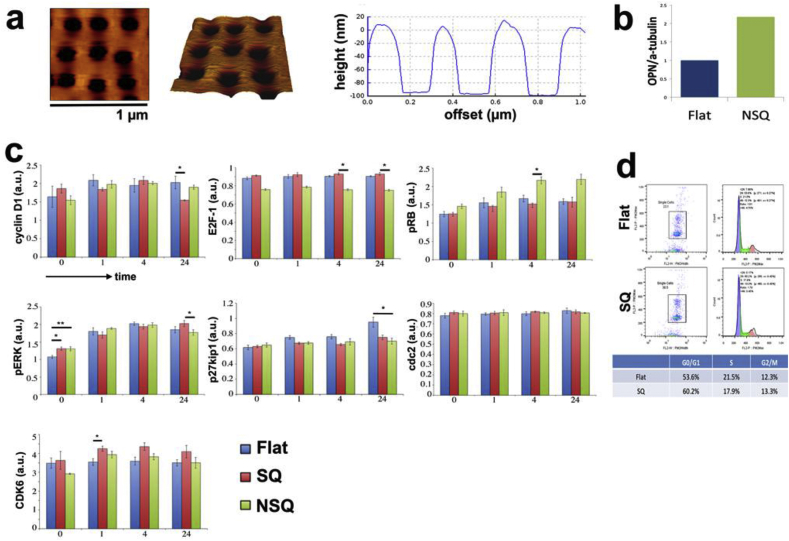
Cell cycle regulation on nanotopography. (a) AFM image representative of the surface of our osteogenic differentiation nanopattern, NSQ. Dark regions indicate the location of the pits, which have a degree of offset (±50 nm) in comparison to the ordered SQ nanotopography. The graph to the right of the images gives an indication of the pit depth and spacing. Scale bar: 1 μm. (b) Verification of the effects of NSQ on SSCs was tested by culturing CD271^+^ SSCs for 28 days and western blot analysis. Results shown are relative OPN protein levels normalised to alpha tubulin (expressed in a.u.). It can be seen that OPN is higher on NSQ, suggesting that osteogenesis is taking place as expected. (c) Synchronised and subsequently released SSCs were assessed for changes in levels of cell cycle-related proteins. Graphs represent mean fluorescence intensity (a.u.) values ± SEM for the protein of interest, normalised to either total protein (pRb to total Rb, pERK to total ERK) or GAPDH (n ≥ 4). Significant changes were observed in cyclin D1, E2F-1, pRb, pERK and CDK6, suggesting that cell cycle regulation is altered by nanotopography. *p < *0.05,* **p < *0.01*, by ANOVA. (d) Flow cytometry data illustrating an increased preponderance for SSCs to be in G0/G1 phase on SQ (n = 2).

Satisfied that the nanotopography exerts self-renewal effects on multiple SSC sub-populations, we moved back to use of STRO-1 populations, as are routinely used in our labs, for investigation of biological mechanism. In the introduction, we hypothesised that self-renewing SSCs will undergo changes in cell cycle regulation, potentially through changes in MAPK signalling. To study this we examined expression levels and activation of mediators of cell cycle progression. In this section, we introduce an extra control in addition to the flat surfaces. We employ our near-square (NSQ) surface as a positive osteo specific differentiation control [7], [17], [27]. This topography is similar to the SQ surface but with an up to ±50 nm offset from the centre position (Fig. 3a). Briefly, the surface was tested for osteogenesis by western blot densitometry for osteopontin (OPN, a bone specific marker) after 28 days of STRO-1 culture (seeded at 1000 cells/cm^2^) and showed increased expression compared to SSCs on flat control or the SQ pattern (Fig. 3b) confirming published data [7], [17], [27]. Use of NSQ allowed us to compare and contrast growth between three distinct groups: SSC self-renewal on SQ, typical culture phenotypical drift during proliferation on flat controls and targeted osteoblastic differentiation on NSQ.

Protein-level quantification of expression and activation of a range of cell cycle-related proteins was next undertaken (Fig. 3c). Following serum starvation to hold cells at G0/G1 [28], [29], [30] (SSCs were seeded for 24 h in 10% FBS containing media before switching to 0.1% FBS for a further 48 h), release of synchronised SSCs back into the cycle (reversion to 10% FBS containing media) for 1, 4 and 24 h, revealed significant differences in levels of progression regulatory proteins including positive regulators of cycle progression - E2F transcription factor 1 (E2F-1) and cyclin D1 as well as negative regulators - phosphorylated retinoblastoma protein (pRB) and p27^kip1^.

Increased phosphorylated retinoblastoma protein (pRB) levels were consistently observed in cells on NSQ while levels remained low on both the control and SQ. E2F-1 protein levels were seen to reduce at later time-points on NSQ (Fig. 3c). Levels of the cyclin-dependent kinase inhibitor, p27^kip1^, were also noted to be significantly lower in cells on NSQ (Fig. 3c) in comparison to cells on flat control and SQ. For SSCs on SQ, the CDK6 protein level was seen to be elevated and cyclin D1 levels lowered compared to SSCs on control or NSQ (Fig. 3c).

As the majority of changes in cell cycle proteins between surfaces were observed after 24 h of progression from re-entering the cell cycle, we performed flow cytometry for SSCs cultured on SQ compared to SSCs on flat control at this time point. Analysis revealed an increased trend for SSCs to remain in G0/G1 when cultured on SQ (from 53.6% on flat to 60.2% on SQ - Fig. 3d and Supplementary Fig. 1 for larger view of the flow analysis).

Our data also shows changes in ERK 1/2 activation (increased phosphorylation on both surfaces) and after 0 and 24 h with decreased activation on NSQ (Fig. 3c).

### 3.3 Transcriptional analysis of SSC self-renewal

Results demonstrate that t24 h post synchronisation was associated with significant changes in the expression of cell cycle regulatory proteins (Fig. 3c). We therefore performed next generation sequencing (RNAseq) transcriptional analysis after 24 h to further determine the full range of genes that are regulated during the transition from cell cycle to differentiation. We compared SSCs on SQ (and in supplementary dialogue NSQ) to those on flat control and identified pathways that included hits from cell cycle analysis (Fig. 3) in order to further identify regulators of SSC self-renewal. To confirm and expand this transcriptional analysis, untargeted metabolite analysis using mass spectrometry was also performed after 7 and 28 days of culture (we chose longer, un-synchronised time points for metabolomic analysis as short term metabolite abundance changes can be small) [27].

**Fig. 4:**
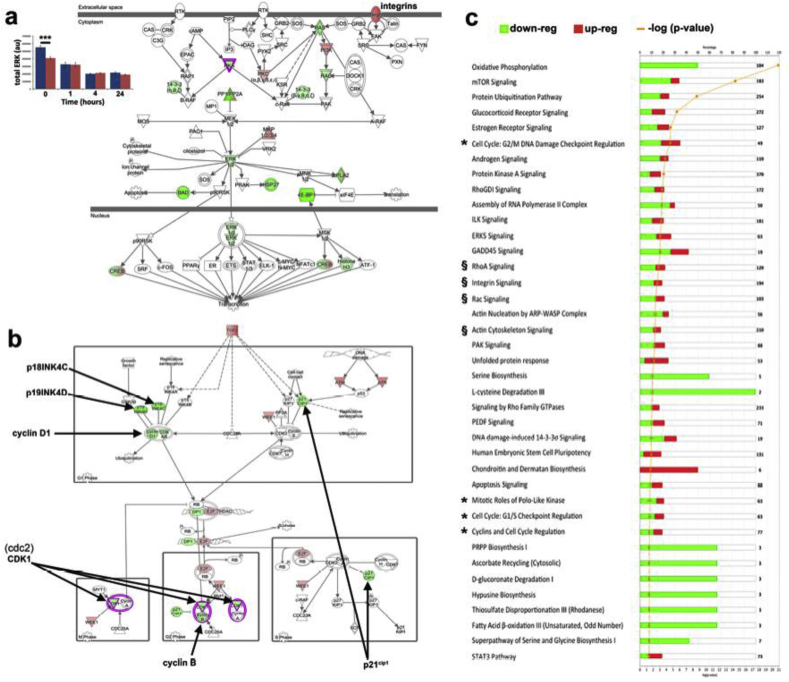
Mechanisms underlying SQ induced self-renewal. (a) Graph (inset) shows in cell western results for total ERK 1/2 for SQ (red) and flat (blue) across 0, 1, 4 and 24 h. ERK 1/2 was consistently lower on SQ when compared to the control (significantly at t = 0), and decreased progressively when moving through the cell cycle. ***p < 0.001, t-test. RNAseq analysis generated a network in IPA that links ERK 1/2 and adhesion-based signalling on SQ 24 h following release into the cycle after synchronisation. ERK 1/2 was also indicated to be down-regulated here. (b) Several genes associated with cell cycle progression (CDK1 (cdc2), cyclin B, cyclin D1) and those related to cycle repression (p18INK4C, p19INK4D and p21^cip1^) were up-regulated (green) on in cells on SQ (arrows point to all named genes). (c) Canonical pathway analysis underlines differential regulation of cell cycle (denoted as *) and adhesion (denoted as §) in cells on the SQ topography compared to those on control. A general trend of down-regulation can be observed across the majority of signalling groups for cells on on SQ. Note that only pathways with *p* < 0.05 are shown. A two-fold change cut-off was applied for RNAseq data (n = 3). (For interpretation of the references to colour in this figure legend, the reader is referred to the web version of this article.)

Two gene networks were identified by RNAseq that included hits for cell cycle regulators. Firstly, Fig. 4a implicates cell adhesion in the negative regulation of ERK 1/2 in SSCs on SQ compared to flat control. Measurement of total ERK 1/2 protein levels showed that levels are suppressed in cells on SQ at t = 0 compared to SSCs on flat control (Fig. 4a inset). However, this did recover when cells were released back into the cell cycle (Fig. 4a inset). We note that the MAPK Jnk was a more highly ranked network for SSCs on NSQ and that Jnk was positively regulated (Supplementary Fig. 2). The amino-acid metabolite network shown in Fig. 2b indicated negative regulation of ERK 1/2 was likely; the same network for SSCs on NSQ predicted positive regulation (Supplementary Fig. 3).

RNAseq analysis indicates cyclin D1, cyclin B and cdc2 (cell division cycle 2 or cyclin dependant kinase 1, CDK1), positive regulators of cell cycle progression, become down-regulated on SQ (Fig. 4b). The same analysis also indicated down-regulation of a number of negative cell cycle regulators - *p21*^Cip1^, *p19INK4D* and *p18INK4C*) in SSC on SQ (Fig. 4b). This transcription-level repression of cyclin D1 in SSCs on SQ was also reflected at protein level (Fig. 3c) while we note that the down-regulation of cdc2 seen in Fig. 4b was not seen at protein level (levels were unchanged, Fig. 3c).

Canonical pathway analysis revealed that a number of significant pathways are implicated in cell cycle control, such as G1/S checkpoint regulation, cyclins in cell cycle regulation, G2/M checkpoint regulation and mitotic role of polo-like kinase – it is noteworthy that these have balanced up-and down-regulation (Fig. 4c – denoted by *). For SSCs on NSQ, many more pathways were highlighted as significant (Supplementary Fig. 4). Again, the same cell cycle pathways were noted and, again, they were shown to have a balance of up and down-regulation (Supplementary Fig. 4). Further, components of adhesion-related signalling processes including RhoA, integrin, Rac, and actin cytoskeleton signalling were implicated as differentially regulated compared to flat control in cells on SQ (Fig. 4c – denoted by §) and NSQ (Supplementary Fig. 4).

### 3.4 Metabolomic analysis of SSC self-renewal

**Fig. 5:**
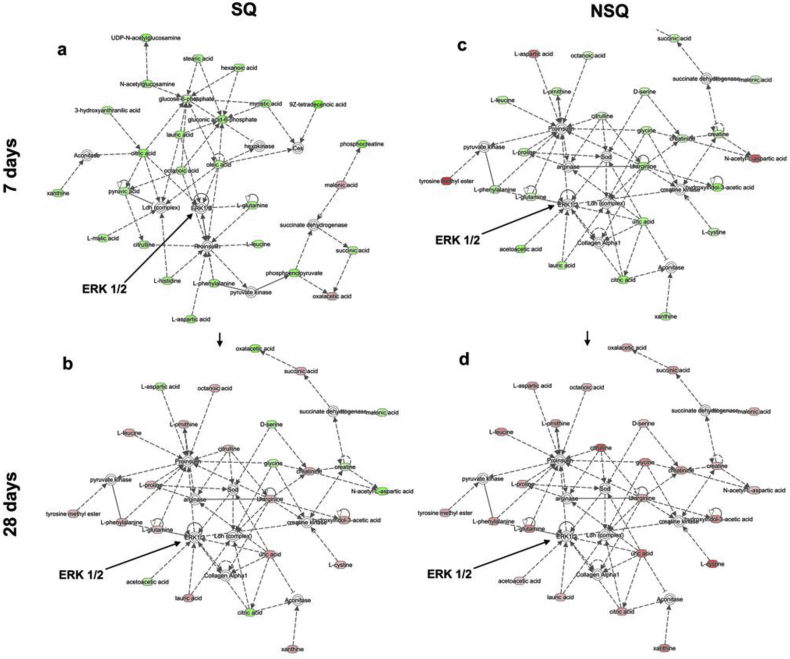
Comparison of amino acid networks generated by metabolic analysis. A common amino acid network with associations to ERK 1/2 was identified on SQ and NSQ in STRO-1^+^ SSCs after 7 and 28 days of culture. (a) After 7 days, a high degree of down-regulation (green) was apparent on SQ with (b) some up-regulations (red) being observed after 28 days (c) at 7 days on NSQ more amino acids were up-regulated (red) than SQ. (d) this trend became more obvious after 28 days, where the majority of amino acids linked to ERK 1/2 were up-regulated (n = 3). (For interpretation of the references to colour in this figure legend, the reader is referred to the web version of this article.)

As has been described, changes in MAPKs will be driven by changes in the levels of metabolites [7]. Here, we move to confirm and expand on data in Fig. 2 (derived from CD271^+^ SSCs) with STRO-1^+^ SSC populations after 7 and 28 days of culture. Analysis of metabolites highlighted an amino acid network common to SQ and NSQ (Fig. 5) linking to ERK1/2. The networks are revealing as on SQ at 7 days they showed strong down-regulation of metabolites linking to ERK 1/2 (Fig. 5a) while on NSQ a number of up-regulations were apparent (Fig. 5b). By day 28, while there were a number of up-regulations now apparent in SSCs on SQ (Fig. 5c), all the metabolites linking to ERK 1/2 were up-regulated on NSQ (Fig. 5d).

## 4 Discussion

The current study examined the importance of cell cycle control in the regulation of SSC self-renewal and multi-potentiality. First, we noted that not all SSCs are the same, for example while STRO-1^+^ SSCs retain STRO-1 expression on the SQ surface when seeded at high or low density, CD271^+^ cells required a stringent, low-density, seeding protocol to allow CD271 retention during cell expansion. That SSCs are heterogeneous is not a new observation, but clearly important when considering technologies that need to span different labs with different isolation protocols or indeed that utilise commercially available SSCs. In our hands, CD271^+^ SSCs were less sensitive to the surfaces than STRO-1^+^ SSCs. To achieve self-renewal control of both SSC sub-populations we note that the seeding density is critical. At sub-optimal cell densities, where purely clonal growth is seen irrespective of the surface the SSCs are cultured on, underlying topography is not important. If the density is too high, then uncontrolled growth and phenotypical drift is noted. However, there is a ‘Goldilocks’ zone where the nanotopography can provide strong influence over the self-renewal of CD271 isolated cells *and* the STRO-1 isolated cells.

We hypothesised that MAPKs may act as the decision switch between self-renewal and differentiation. Differences in ERK 1/2 phosphorylation were noted for SSCs on SQ and NSQ relative to control. This was supported transcriptionally, where adhesion signalling linked to ERK 1/2 and changes in integrin, G-protein and cytoskeletal signalling were noted. This is in agreement with reports on adhesion and ERK 1/2 being important in osteogenesis [5], [6], [15], [17]. Further, using bioinformatics to link metabolite data to biochemical pathways allows suggestion that SSCs were still ‘working’ to repress ERK 1/2 signalling on SQ even after a month of culture. However, on NSQ, the metabolite signals into the ERK 1/2 cascade were highly up-regulated and, as noted, up-regulation of ERK 1/2 is widely observed in osteogenesis [5], [7], [31].

Investigating cell cycle, increased pRB levels were noted in SSCs on NSQ, suggesting early cell-cycle repression at the transition between G1 and S phase as RB phosphorylation blocks S phase entry. Accumulation of pRB protein may be beneficial during the switch from proliferation to differentiation as pRb has been reported to interact with transcription factors specific to osteogenesis and adipogenesis (Runx2 and PPARγ) [32]. It can therefore be postulated that higher levels of pRb may influence the promotion of osteogenesis. The E2F-1 transcription factor, which acts as a molecular ‘switch’ between proliferation and differentiation [33] can bind pRB. Unbound, E2F-1 promotes G1 to S phase transition. However, when the pRB/E2F-1 complex is formed the G1 to S phase transition is inhibited [34]. It is thus noteworthy that the decrease in E2F-1 protein levels at later time-points on NSQ thus correlates with the observed increases in pRB levels.

Down-regulation of p27^kip1^ in SSCs on NSQ indicates that the SSCs are not completely stopping cell cycle progression as targeted osteogenic differentiation is induced by NSQ. Rather, this may suggest a more subtle mechanism to slow cell growth, as it typically seen with osteoblastic differentiation, with some negative regulators up-regulated (*e.g.* pRB) and others down-regulated (*e.g.* p27^kip1^).

For self-renewing SSCs on SQ, cell cycle regulation was also seen to change. High CDK6 protein levels in HSCs correlates with exit of quiescence following mitogenic stimulation [35] and, interestingly, overexpression of CDK6 has been shown to block BMP-2 induced osteoblast differentiation [36]. Our data shows that CDK6 levels were elevated on SQ and while we can postulate that this increase in CDK6 may be involved in preventing differentiation, we note that the observed increase was modest and we therefore hypothesise that an intermediate level of CDK6 is important for self-renewal, allowing the SSCs to increase in number steadily, but not to undergo differentiation.

Repression of cyclin D1 in SSCs on SQ concurs with the noted increase in G1 preponderance as it is required to form a complex with *e.g.* cdc2 (cell division cycle 2) to drive cell cycle progression. We note that levels of cdc2 (CDK1) were relatively similar on all surfaces (Fig. 3c).

Together, our protein-level data suggests that different modes of cell cycle control select between self-renewal (SQ) and active differentiation (NSQ) in SSCs. While self-renewing SSCs appear to hold in G1 via a cdc2 dependant mechanism, osteo-committed cells are shown to repress S phase transition via p27^kip1^. Further, changes in ERK1/2 regulation in SSCs on the surfaces helps identify this MAPK as a potential cell-cycle regulating switch for self-renewal and differentiation in SSCs. Supported by RNAseq and metabolomics data this ties in with the literature describing ERK and Jnk up-regulation/activation as being important in osteogenesis from SSCs [5], [6], [7], [31].

RNAseq data helps support hypotheses drawn from protein-level data. Cyclin D1, cyclin B and cdc2 (CDK1) are involved in cell cycle regulation during G1 and G2/M phases [37] and have been reported to help regulate pluripotency in human embryonic/induced pluripotent stem cells [38] and were down-regulated in SSCs on SQ. Negative regulators of the cell cycle, *p21*^Cip1^, *p19INK4D* and *p18INK4C*), were also down-regulated on SQ, which suggests that cell cycle progression is occurring on this surface (*i.e.* inhibitors of the cell cycle are not preventing SSC proliferation) but that the cycle is differentially regulated. Again, negative regulators, such as p27^kip1^, are thought to be important for pluripotency of human embryonic stem cells (ESCs) [39] although it should be noted that ESCs have short G1 phases [40], [41], [42].

Transcript analysis also implicated adhesion and cytoskeleton as important in self-renewal (SQ) and osteocommitment (NSQ). This is in line with studies proposing that self-renewing stem cells maintain an intermediate tension phenotype [43], in contrast with the requirement of high tension and well spread morphology in osteoblasts and low tension and rounded/less pronounced cytoskeletal changes for adipogenesis [44], [45].

Observations of the levels of cell cycle control proteins provided evidence that on the SQ, self-renewal promoting, substrate, SSCs differentially regulate cell cycle progression with cycle control becoming tightly regulated and a greater preponderance for SSCs remaining in G1. This is in disagreement with reports on pluripotent stem cells [40], [41], [42], but in agreement with reports on adult neural stem cells and suggests cell cycle control [46].

This report helps to highlight differences between *in vitro* renewing SSCs and SSC populations undergoing typical phenotypical drift during culture. While the difference between adipocytes and osteoblasts are large, the differences between SSCs and fibroblasts are subtle but, nonetheless, important. Previous work has highlighted that a slight reduction in adhesion and intracellular tension are required for the SSC phenotype [7]. Our metabolomic analysis showed commonality of mechanism for CD271 and STRO-1 enriched populations with MAPKs, most notably, ERK 1/2 being a target of metabolite control; RNAseq and protein-level data confirmed this. Furthermore, RNAseq transcriptomics data indicated ERK1/2 regulation had contributions from cell adhesion signalling.

Together, this data indicates SSC phenotype is regulated by adhesion, which is a reasonable assumption given that SSCs adhere to the extracellular matrix within their *in vivo* niche. Downstream of the cell adhesions, we hypothesise that ERK1/2 helps regulate SSC cell cycle control to retain stem cell phenotype.

SSCs possess the ability to both self-renew and differentiate, with the maintenance of self-renewal being prominently observed in their niche environment. As our understanding of differentiation mechanisms has progressed, the study of self-renewal has been somewhat limited in comparison. However, the ability to control self-renewal using nanomaterials provides new hopes for SSCs in therapeutic application through the ability to continuously expand SSCs and demonstrates the large potential of nanomaterials as tools for use in cell biology.

## 5 Conclusions

The study demonstrates that cell seeding is of great importance in SSC based experiments. Different labs have different seeding protocols and cell counting suffers inherent variability. As SSC directing materials become more widely researched it is important that labs ensure that their seeding protocol is adapted to get best results from the materials they are generating or adopting.

The study shows that a MAPK switch driving changes in cell cycle regulation to control phenotype likely controls SSC self-renewal and differentiation. This appears sensible as the SSC niche control quiescence, symmetrical and asymmetrical self-renewal to respond to regenerative demand.
