# Supplementary material for: Nanotopography controls cell cycle changes involved with skeletal stem cell self-renewal and multipotency
Source: Biomaterials. 2017 Feb;116:10–20. doi: 10.1016/j.biomaterials.2016.11.032 (PMC5226065; doi:10.1016/j.biomaterials.2016.11.032)
Supplement: Supplementary file 1 [file mmc1.docx]

Supplementary Data

Nanotopography Controls Cell Cycle Changes Involved With Mesenchymal Stem Cell Self-Renewal And Multipotency

Louisa C.Y Lee, Nikolaj Gadegaard, María C. de Andrés, Lesley-Anne Turner, Karl V. Burgess, Stephen J. Yarwood, Julia Wells, Manuel Salmeron-Sanchez, RM Dominic Meek, Richard O.C Oreffo, Matthew J. Dalby*


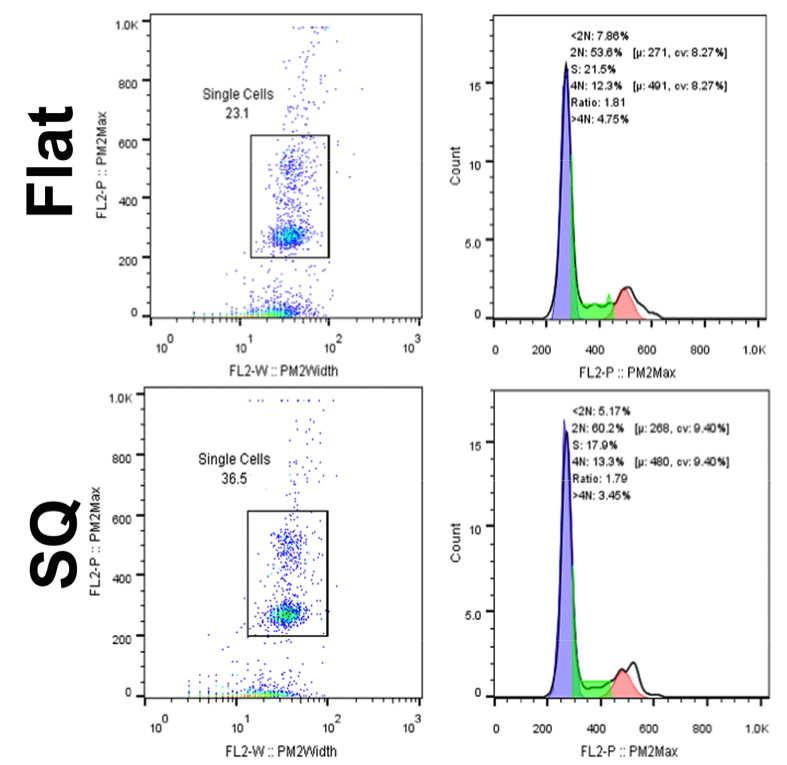


**Supplementary figure 1.** Enlargement of figure 3d to show flow cytometry gating.


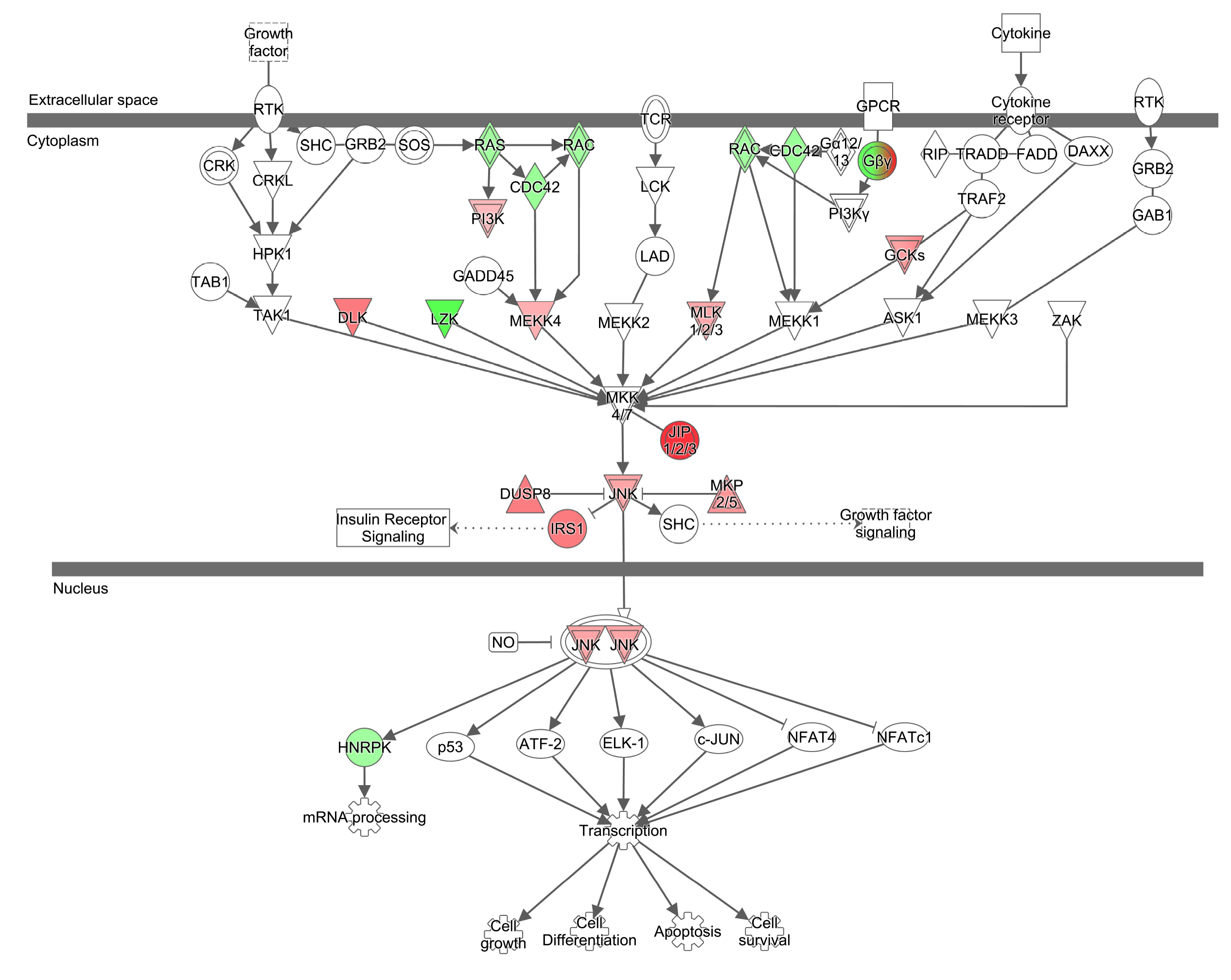


**Supplementary figure 2.**  *Jnk* is up-regulated on NSQ. IPA-generated pathway based on metabolomics data analysis, depicting stress-activated protein kinases (SAPK)/Jnk signalling on NSQ following 24 hours release of STRO-1^+^ SSCs into the cell cycle (SSCs were initially synchronised). *Jnk* was up-regulated on NSQ, which has been implicated in differentiation. A two-fold change cut-off was applied (n=3).

_
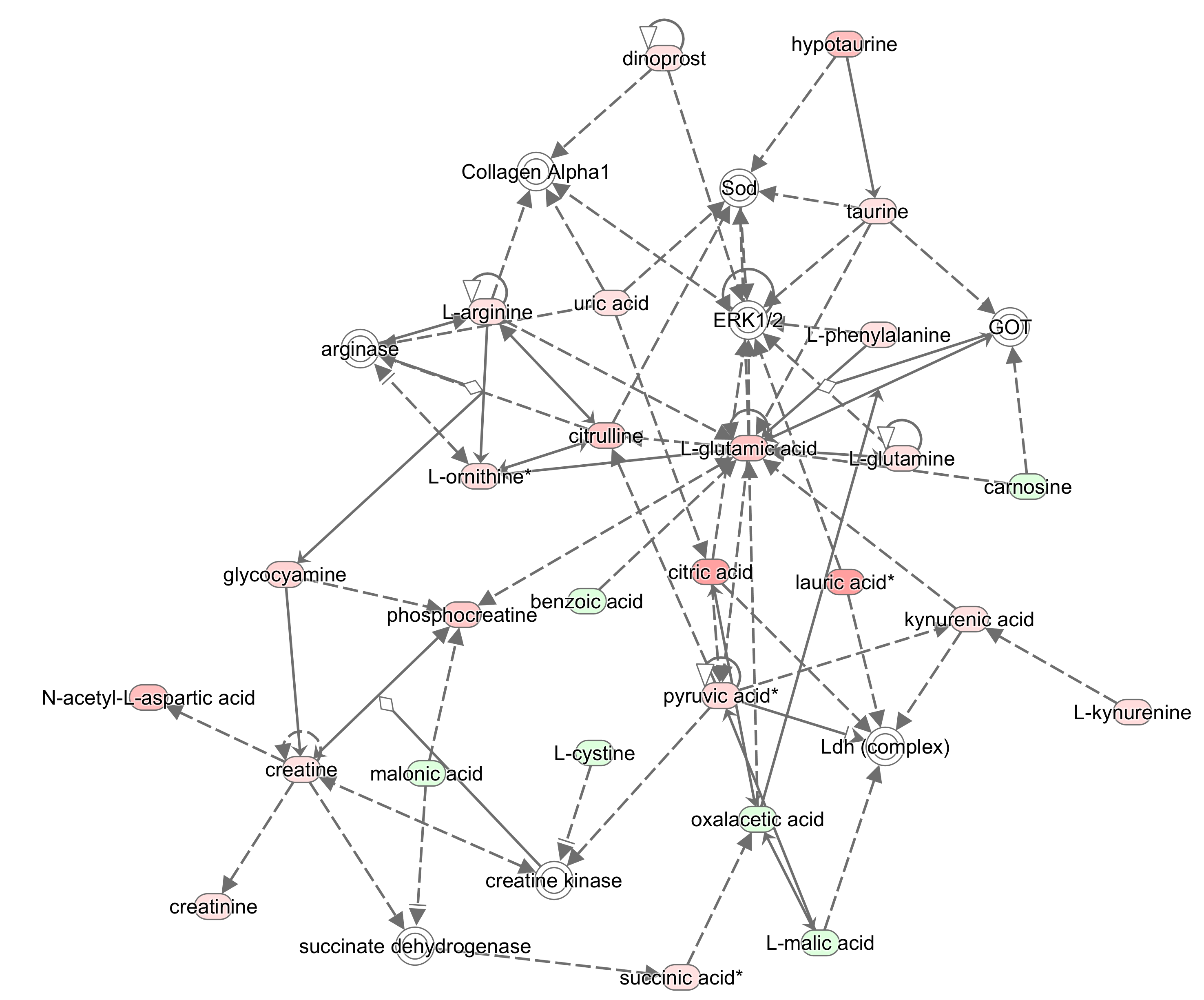
_

**Supplementary figure 3.** Amino acid up-regulation in CD271^+^ SSCs on NSQ. Network was generated in IPA. Metabolomics data from 7 day cultured CD271^+^ SSCs indicated up-regulation (red) of several amino acids with associations to ERK 1/2. This was similar to observations from STRO-1^+^ SSCs (figure 5). (n=6).

_
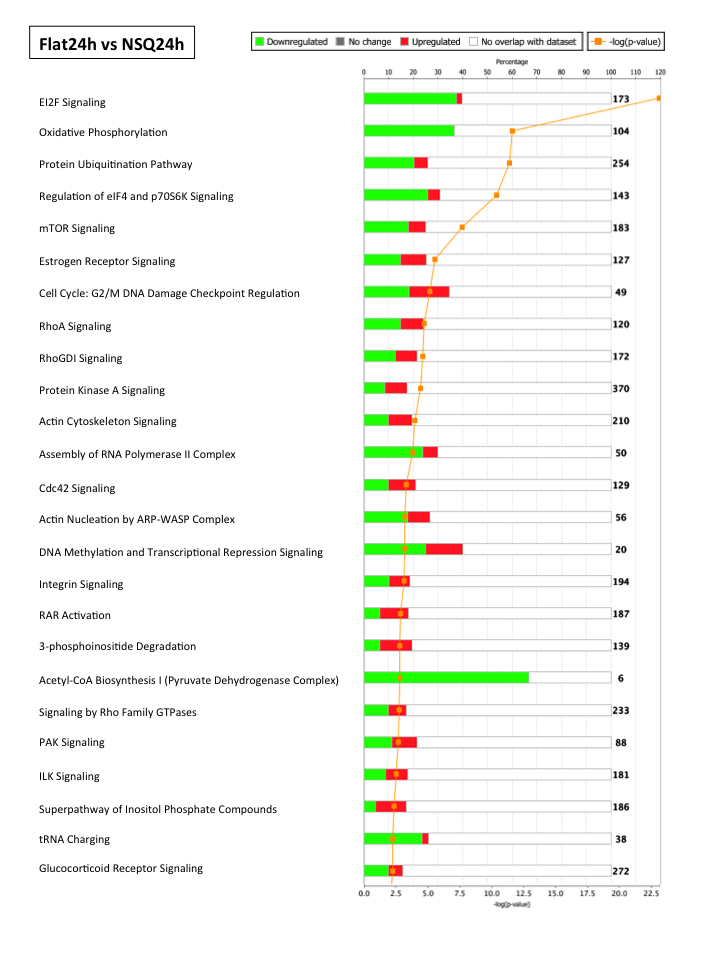
_

_
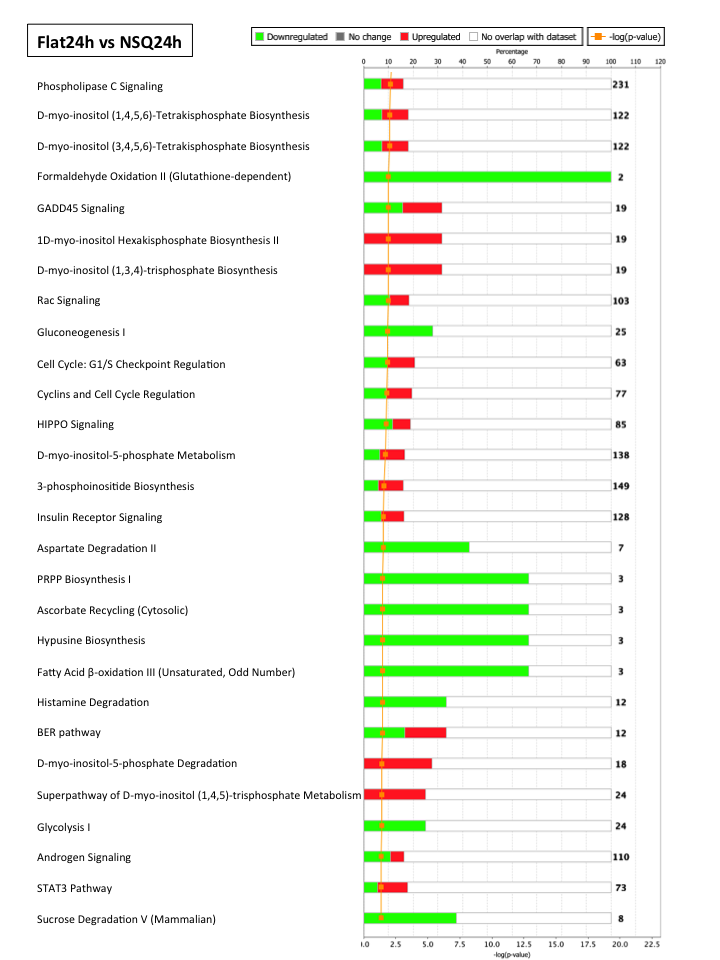
_

**Supplementary figure 4.** Canonical signalling based on NGS data for STRO-1^+^ SSCs that had been synchronised (for 48 hours) and released into the cell cycle (for 24 hours). Values were expressed relative to flat controls. A greater number of up-regulations were identified on NSQ in comparison to SQ. (n=3).
